# Exploring consumer opinions on the presentation of side‐effects information in Australian Consumer Medicine Information leaflets

**DOI:** 10.1111/hex.12215

**Published:** 2014-06-06

**Authors:** Vivien Tong, David K Raynor, Susan J Blalock, Parisa Aslani

**Affiliations:** ^1^ Faculty of PharmacyThe University of SydneySydneyNSWAustralia; ^2^ School of HealthcareUniversity of LeedsLeedsUK; ^3^ Eshelman School of PharmacyUniversity of North Carolina at Chapel HillChapel HillNCUSA

**Keywords:** consumer opinions, risk communication, side effects, written medicine information

## Abstract

**Background:**

Consumer Medicine Information (CMI) is a brand‐specific and standardized source of written medicine information available in Australia for all prescription medicines. Side‐effect information is poorly presented in CMI and may not adequately address consumer information needs.

**Objective:**

To explore consumer opinions on (i) the presentation of side‐effect information in existing Australian CMI leaflets and alternative study‐designed CMIs and (ii) side‐effect risk information and its impact on treatment decision making.

**Design:**

Fuzzy trace, affect heuristic, frequency hypothesis and cognitive‐experiential theories were applied when revising existing CMI side‐effects sections. Together with good information design, functional linguistics and medicine information expertise, alternative ramipril and clopidogrel CMI versions were proposed. Focus groups were then conducted to address the study objectives.

**Participants and setting:**

Three focus groups (*n* = 18) were conducted in Sydney, Australia. Mean consumer age was 58 years (range 50–65 years), with equal number of males and females.

**Results:**

All consumers preferred the alternative CMIs developed as part of the study, with unequivocal preference for the side‐effects presented in a simple tabular format, as it allowed quick and easy access to information. Consumer misunderstandings reflected literacy and numeracy issues inherent in consumer risk appraisal. Many preferred no numerical information and a large proportion preferred natural frequencies.

**Conclusions:**

One single method of risk presentation in CMI is unable to cater for all consumers. Consumer misunderstandings are indicative of possible health literacy and numeracy factors that influence consumer risk appraisal, which should be explored further.

## Introduction

Medicine information helps increase consumer understanding, address consumers' information needs and assist in informed decision making.^1^, ^2^ Consumers have considered written medicine information (WMI) as an important and reliable source of medicine information.^3^ Written resources may help to reinforce medicine information that is difficult to recall, such as side‐effects and dosages.^4^

Consumers seek side‐effects information, alongside other information critical for safe medicines use.^5^, ^6^, ^7^ There is therefore a need for detailed, comprehensive side‐effects and medicine safety information to be made available for consumers at a level that they can understand.^8^, ^9^ Specifically, side‐effects and the likelihood of experiencing a side‐effect is highly coveted by consumers,^10^, ^11^ where the need for their inclusion in WMI is recognized.^12^ Previous work has noted that consumers prefer side‐effects to be categorized according to likelihood and severity^12^, where some have felt that side‐effects information presented in its entirety will help facilitate informed treatment decision making.^8^

Numerical and verbal descriptors can be used to present side‐effect risk information in WMI. Risk overestimation has been associated with verbal descriptor use (e.g. common and rare),^13^, ^14^, ^15^ where there is room for error when consumers translate this information into numerical terms.^16^ Numerical descriptors have been favoured for the use in WMI,^12^ where absolute (natural) frequencies (e.g. three in 100 people will experience a side‐effect) has led to increased consumer satisfaction in comparison with verbal descriptor use^17^ and have on the whole supported more accurate consumer risk estimates compared with frequency bands (e.g. less than 1 in 100 people but more than 1 in 1000 people will experience a side‐effect).^18^ However, consensus has not been reached on a superior numerical descriptor that promotes accurate consumer understanding. A similar proportion of accurate consumer side‐effect risk estimates were seen, when presented as either percentages or frequencies.^19^

Benefit–risk information may lead to treatment reservations for some and empowerment in treatment decision making for others.^20^ Consumer appraisal of benefit–risk information thereby plays an important role, as it can impact intended treatment decision making.^21^ The impact of risk information presentation (such as framing effects demonstrated through positive framing use, which fuelled consumer preference for a particular treatment^22^) coupled with consumer overestimation of risk^16^, ^19^, ^23^ highlights the complexities of side‐effect risk presentation in WMI.

Side‐effect risk information appears to have been poorly presented in a proportion of WMI.^24^ Moreover, studies have explored the use of risk descriptors (as isolated text separate to other text normally found in WMI) without contextualization within WMI as a complete medicine information source,^15^, ^16^, ^18^, ^19^, ^23^, ^25^, ^26^ signalling the need for further work. Moreover, psychological models relevant to risk communication and perception remain neglected when reformatting and revising WMI.

In Australia, there is a legal requirement for pharmaceutical manufacturers to supply Consumer Medicine Information (CMI) with all prescription and pharmacist‐only medicines (over the counter medicines that are required to be handed out by the pharmacist).^27^ CMI are brand‐specific and standardized sources of WMI, whose content is guided by legislation. They are used by consumers as an important source of medicine information.^5^, ^7^ However, there are limited Australian studies that have sought to discern consumers' views specifically on side‐effects information in CMI leaflets. Therefore, this study aimed to explore consumers' opinions on (i) the presentation of side‐effects information in Australian CMI leaflets and (ii) side‐effect risk information and its impact on their treatment decision‐making processes.

## Methods

This study comprised of two stages:

1. Reformatting and revising the CMI side‐effects sections for ramipril (Tritace^®^ brand; Sanofi‐Aventis Australia Pty Ltd, Macquarie Park, Australia) and clopidogrel (Plavix^®^ brand; Sanofi‐Aventis Australia Pty Ltd, Macquarie Park, Australia).
2. Qualitative exploration of consumer opinions, understanding and decision‐making processes relating to side‐effects and side‐effect risk information.

### Stage 1: Reformatting and revising the CMI side‐effects sections

#### Psychological model selection

Appropriate psychological models acted as theoretical frameworks underpinning the reformatting and revising of the side‐effects section of the existing (original) CMI for ramipril (last revised 2007 by the pharmaceutical company/sponsor) and clopidogrel (last revised 2009), postulated to help promote accurate consumer risk appraisal and account for subjective risk perception.

Models were selected according to their appropriateness of application to WMI and relevance to risk perception and communication. Fuzzy trace,^28^, ^29^ affect heuristic,^30^, ^31^, ^32^ frequency hypothesis^28^ and cognitive‐experiential^33^ theories were chosen. In conjunction with the principles of good information design, functional linguistics and medicine information expertise,^34^ four alternative CMI versions were produced for each study medicine (Table 1).

**Table 1:** Summary of the components of each alternative CMI version

Version (V)	Models/theories	Framing	Numerical descriptor	Other changes	Examples of key changes
V1	None	None	None	Application of good information design, functional linguistics and medicine information expertises^34^ (V1 was the foundation for other alternative CMIs)	Two‐column format (compared with three‐column format of existing CMI) Side‐effects tabulated in separate tables with two columns based on severity (e.g. very serious/serious/mild) and action to be taken if side‐effect occurs, that is, one column lists the side‐effects, and the second column lists the action to be taken (e.g. contact the doctor)
V2	Fuzzy trace theory Affect heuristic	Positive	Percentages	Side‐effects alphabetized^a^ Benefit information included	Side‐effects categorized based on action to be taken and then tabulated in separate tables with two columns, based on severity (e.g. very serious/serious/mild), that is, one column lists the side‐effects, and the second column lists the likelihood of side‐effects Percentages and verbal descriptors (where possible) were used to show likelihood of NOT experiencing a side‐effect (positive framing), for example, 98% and more (less common) Benefit information about the medicine was included
V3	None	Positive	Percentages	Side‐effects alphabetized^a^	Similar to V2, but without the inclusion of benefit information
V4	Frequency hypothesis Cognitive‐experiential theory	Negative	Natural frequencies	Side‐effects alphabetized^a^	Tabulation of side‐effects as in V2 Natural frequencies and verbal descriptors (where possible) were used to show likelihood of experiencing a side‐effect (negative framing), for example, two in 100 and less (less common); three in 100 No benefit information was included

a Formatting changes not informed by theory.

#### Selected models and considerations

Selected models were used in combinations to support consumer understanding, where each had certain strengths and weaknesses when applied to side‐effect risk communication in WMI (Table 2).

**Table 2:** Strengths and weaknesses relevant to the model application in the CMI reformatting and revising process

Model/theory	Description of model/theory	Strengths	Weaknesses
Fuzzy trace (FTT)	Dual‐process theory comprising two information representations: verbatim (literal aspect) and gist (interpretation or understanding of presented information)^28^, ^29^ Consumers encode information using both gist and verbatim representations; gist representation is emphasized as the advanced encoding process	Established extrapolation to health risk communication and perception^28^, ^29^ Pertinent to medical decision making^29^	Does not provide as much detail regarding a preferred numerical descriptor to communicate risk and help consumers perform gist encoding
Affect heuristic (AH)	Subjective responses are critical to decision making^30^, ^31^, ^32^ Perceived benefit and risk are intrinsically linked	Able to account for subjective perception of risk^30^, ^31^, ^32^	Provides rationale for inclusion of benefit information, but unable to provide a detailed framework for the reformatting and revising of side‐effects sections on its own
Frequency hypothesis (FH)	Computational approach,^28^ supportive of reduced computations required to be performed by a consumer to gauge meaning Predicts natural frequencies are easier to understand as consumers have direct experience with them	Can be used to select a numerical risk descriptor (natural frequencies) which can theoretically aid consumer understanding^33^	Does not necessarily provide detailed projections regarding consumer decision‐making processes
Cognitive‐experiential theory (CET)	Recognizes individual differences inherent in information processing^33^ When applied, CET suggests preferential consumer bias towards smaller numbers (denominators) due to prior experience	Accounts for individual differences in the processing of information^33^ Explains favouring of smaller numbers over larger numbers^33^	Limited literature supporting application to health risk communication and in particular to written medicine information

##### Fuzzy trace theory

Percentages were chosen to convey side‐effect risk information, underpinned by fuzzy trace theory (FTT), due to a reported consumer desire for percentages^34^ and risk appraisal accuracy in relation to percentages.^35^ Furthermore, to help promote the intended gist (side‐effects will not be experienced by the majority), positive framing (likelihood of not experiencing a side‐effect) was used in V2 to support consumers' gist formation (Table 1). The intended gist was proposed to target widespread consumer overestimation of risk associated with verbal descriptor use.^13^, ^14^, ^15^

##### The affect heuristic

Benefit information is expected to impact an individual's subjective view (affect) and therapy decision making. Consumers want positive information provided before side‐effects^9^ as well as benefit–risk information.^12^ Therefore, in V2, both benefit and side‐effect risk information were provided concomitantly (Table 1). The integration of benefit information into the beginning of the side‐effects section was predicted to supplement the positive framing and aid balanced decision making.

##### The frequency hypothesis

As frequency hypothesis (FH) assumes that consumers have direct experience with natural frequencies and predicts ease of understanding in comparison with artificial constructs such as normalized probabilities or decimals,^33^ all probabilities were presented as natural frequencies in V4 (Table 1).

##### Cognitive‐experiential theory

Experience with smaller numbers, as opposed to larger ones (i.e. denominators), indicates that consumers have preferential bias towards smaller numbers. Therefore, when applying FH and cognitive‐experiential theory (CET) to V4, frequencies were expressed using the smallest denominators, where possible (Table 1). However, large denominators were unavoidable and have been utilized where smaller denominators were not meaningful.

#### Sourcing and use of numerical side‐effect risk information

Numerical side‐effect risk information for clopidogrel was sourced from the CAPRIE study data presented in the Australian clopidogrel Product Information (PI).^36^ Due to limited numerical data in the Australian ramipril PI, relevant figures were extracted for use from the FDA ramipril (Altace^®^ brand) prescribing information.^37^

All numerical figures identified and used were rounded to the nearest whole number. Side‐effects that had no distinct numerical risk estimate were matched to the verbal descriptor used to describe their likelihood in the PI. The numerical range that defined the corresponding descriptor was then included in the reformatted and revised side‐effects sections where appropriate (e.g. ‘less common’ equated to 2% and less, which was then reported for that specific side‐effect). ‘Likelihood unknown’ was quoted when numerical side‐effect risk information was unavailable or could not be matched to a side‐effect in the PI.

### Stage 2: Qualitative exploration of consumer opinions, understanding and decision‐making processes

A qualitative exploration was undertaken to address the study objectives.

#### Choice of qualitative method

Focus groups were chosen because they can help clarify concepts and promote the generation of hypotheses and are useful when examining consumer perceptions, attitudes and behaviour.^38^ The group dynamic may be conducive to the conception of novel insights.^39^

#### Focus group participants and logistics

Participants were recruited using a market research company, where potential participants were identified from their consumer database to meet the study inclusion criteria. A recruitment brief, outlining the project objectives and inclusion criteria, was provided to the company to guide the recruitment process. Participants had to be as follows:

1. Aged 50 years or above.
2. Currently taking at least one prescription medicine or have taken so in the last 6 months.
3. Able to participate without needing a translator.
4. Not currently taking the study medicines (ramipril and clopidogrel).

Ramipril and clopidogrel CMIs were provided to participants 1–2 weeks prior to the focus groups. Participants were provided with the participant information statement and consent form on the day, in addition to a demographic questionnaire. Consumers were compensated $50 (Australian Dollars) for their participation.

Three focus groups of six participants (total 18 participants) lasting 1–1.5 h were conducted in Sydney, Australia (theoretical saturation of ideas^40^ was attained by the third focus group). Mean participant age was 58 years (range 50–65 years), with equal number of males and females. English was the main language spoken at home, with the majority of participants attaining a Higher School Certificate (year 12) or lower level of education.

#### Group discussion protocol and data analysis

Discussions were structured to explore consumer thought processes when making therapy decisions in response to medicine information provided. Consumers were asked for their opinions, understanding and decision‐making processes in response to the alternative CMIs presented. Questions were also incorporated to examine each psychological model's appropriateness to actual consumer risk appraisal (as opposed to predicted consumer risk appraisal). Specifically, consumers were asked to explain their understanding of the side‐effect risks described.

Each focus group was audio‐recorded, with the consent of all participants, and transcribed verbatim. Thematic analysis was conducted independently by two researchers, with emergent themes discussed and agreed upon to ensure identification of all themes.

This study received approval from the University of Sydney Human Research Ethics Committee.

## Results

Consumer responses from the focus groups were broadly categorized into the following: (i) findings related to the formatting and layout changes in both existing and alternative CMIs and (ii) consumers' interpretation of the side‐effects information in the original and revised alternative CMIs.

### Consumer perspectives – format and layout

Every consumer preferred at least one of the alternative CMIs to the existing original CMI, particularly the two‐column design (instead of the three‐column design adopted by original CMI). Consumers felt that the small font size used in existing CMI was difficult to read. Furthermore, some consumers also expressed that the CMI content was overwhelming, in part due to long lists of information.

Conversely, consumers liked appropriate bolding for emphasis and bullet points. Consumers preferred side‐effects to be categorized according to severity, where one consumer preferred categorization in order of ascending severity and others preferred similar side‐effects to be grouped together.

There was an overwhelming preference for V1, where consumers found the tabular format more acceptable, simple, understandable and easier to navigate. Consumers noted that it was easier to find the action needed to be taken, highlighted through the tabular format.

I like it because I go straight back to fault finding in car manuals and that's what you've done here. It gives you a problem and how to fix it (Focus Group 1 (FG1), Participant 5 (P5))

Interestingly, consumers did not recognize that the side‐effects were alphabetized or ordered according to likelihood until prompted.

### Understanding of side‐effects information

Consumer understanding of the terminology utilized to convey side‐effects information fluctuated significantly, where particular terms were susceptible to consumer misunderstanding. Problematic terms included side‐effects, likelihood and severity, in addition to ‘likelihood unknown’.

#### Side‐effects

Consumers had difficulty distinguishing between symptoms, side‐effects and allergies, where one consumer commented that the side‐effects ‘yellowing of the skin’ and ‘dry cough’ were an allergy and intolerance, respectively.

Interestingly, side‐effects were not always negative, where side‐effects such as weight loss were deemed favourable, increasing consumers' willingness to take a medicine in light of these ‘positive’ side‐effects. Conversely, high perceived severity resulted in decreased willingness to take the medicine.

#### Likelihood and severity

Consumers were confused and unable to define likelihood and severity.

It says very serious side effect… and then they say 97% (will) not (be) experiencing this side effect. Why would this one be a very serious side effect? (FG2P1)

Likelihood and severity were used interchangeably by consumers. One consumer correlated severity with the action needed to be taken. Another consumer correlated ‘very serious’ side‐effects with a high likelihood, and others believed the opposite. Severity was subjective, where more serious side‐effects increased consumer concern and caused disagreement in the classification of certain side‐effects.

A proportion of consumers did not understand ‘likelihood’. ‘Likelihood’ was interpreted as the time of onset of the side‐effect, where one focus group reached consensus that a dry cough and yellowing of the skin would occur within 12 and 48 h, respectively. Common side‐effects were perceived to have a fast time of onset. When asked, an overwhelming majority was unable to provide a numerical risk estimate for specific side‐effects. However, consumers acknowledged risk variation and the concept of individual risk levels, where side‐effect risk was dependent upon factors like ‘the health level of the person’ (FG1P2) and existing allergies.

#### Likelihood unknown

Consumers were strongly opposed to the use of ‘likelihood unknown’ and regarded it as illogical. Consumers believed that these side‐effects were included due to legal obligation and were pharmaceutical manufacturers' efforts to prevent consumers from seeking legal action if side‐effects were experienced. It caused distrust, where incompetency and the need for further studies were implied.

No way. That [likelihood unknown] shows ignorance and non‐testing. (FG1P4)

When asked what ‘likelihood unknown’ meant, one consumer stated that ‘the likelihood is infinity’ (FG2P6). Conversely, ‘one in a million’ (FG3P5) was suggested by another consumer to represent a small side‐effect risk in relation to ‘likelihood unknown’.

### Understanding and opinions of numerical side‐effect risk information

Diversity in consumer opinions and understanding of numerical side‐effect risk information is a core finding. The use of natural frequencies, percentages and positive framing had varying impacts upon the consumers.

#### Consumer opinions regarding numerical side‐effect risk information

Numerical side‐effect risk information worried some consumers. The presence of numerical information caused distrust and was dismissed as an inaccurate representation of true likelihood of occurrence. Consumers were more focused on determining individual side‐effect risk, as opposed to relying on population side‐effect risk.

I've had two drug reactions and I think really when it happened to me, I didn't think that. As you know, our bodies and our genetics are different. Something in those two things didn't agree with me but it doesn't mean it wouldn't agree with you. (FG3P6)

One consumer questioned the appropriateness of the extrapolation of data derived from testing to the general population, where ‘they might be using a group of 1000 people and it is going out to millions of people’. (FG2P6)

#### Natural frequencies

A large proportion of consumers preferred natural frequencies included in V4. Consumers stated that it was not ‘natural’ to think in terms of percentages, where natural frequencies required less computational processes.

Yeah, I don't like working in percentages. I'm lazy. Just to say three in a hundred suits me. (FG3P2)

Overall, consumers wanted to know the test population size from which the risk values were derived. Natural frequencies were perceived to provide this, as they were easily associated with distinct patient population numbers. Consequently, natural frequencies embodied more certainty and specificity for consumers in comparison with percentages.

Three in 100 is more narrowing (because for) every 100, there is only three. But 3%, it could be as a mass but then they take the average. (FG2P1)

Natural frequencies provided a realistic dimension to risk, where a denominator of 100 was interpreted literally as a small sample size in comparison with the total population. Consumers assumed that the denominator correlated with the fixed sample size from which the information was derived. Similarly, when the risk of a side‐effect was reported as in one in 10 000, a consumer commented:

At least 10 000 [people] have got to be on that drug before they can make a claim like that. (FG3P5)

Furthermore, the numerator was also a source of concern. One consumer (a caregiver of her adult daughter who experienced life‐threatening side‐effects) commented:

Less than one in ten thousand; well that is not much is it? But you wouldn't want to be that one. (FG2P4)

Despite the low likelihood of one in 10 000 experiencing this very rare side‐effect, apprehension about being this one person dissuaded the consumer from taking the medicine.

Overall, consumers agreed that natural frequencies were a more appropriate way to present smaller likelihoods, as whole numerators and denominators were easier to understand, as opposed to a small percentage containing a decimal (e.g. 0.01%). However, larger and non‐uniform denominators utilized in an alternative Plavix^®^ CMI version caused confusion for some consumers.

#### Percentages

Percentages, included in V2 and V3, were preferred by a smaller proportion. Some consumers reported percentages to be easier to understand, whereas others thought the opposite. Percentages indicated to consumers that the sample population size could vary and was perceived to ‘turn us [consumers] into commodities’ (*FG1P2*).

A key finding was consumers' perceived non‐equivalence between numerical risk descriptors. Consumers were unsure of whether it was possible to translate percentages into equivalent natural frequencies accurately. One consumer incorrectly emphasized that percentages were averages and therefore not equivalent to natural frequencies.

It depends if you look at it as an average, an average of 3%. Maybe 200 will only have one (who experiences the side effect) and 100 would have four or five. (FG2P1)

#### Positive framing and numeracy

Positive framing use, as seen in V2 and V3, decreased worry for some consumers whilst others found it disconcerting. Positive framing of numerical side‐effect risk information appeared to increase consumer willingness to take a medicine.

What it [positive framing] does to me is negate all the side effects. There's 99, 99, [and] only one is 92; and so you're not going to get it and people will take it anyway, that's what I think… He's the only one who puts himself in the 3%. 99% of the people think they're normal. (FG3P1)

Interestingly, consumers who could easily interconvert between positively and negatively framed risk information were less susceptible to framing effects. Furthermore, individual perceptions superseded framing effects for some, where one consumer's own perceived individual risk was high regardless of framing.

With my luck, I'll be in the 3% so it doesn't matter. (FG3P5)

Consumers tended to ignore table headings which gave rise to misinterpretation and worry.

When I look at this, I saw the figure and percentage and didn't go on to read it… I saw it as that's your chances of getting it and it should be round the other way. (FG1P5)

Moreover, some found it difficult to relate positively framed side‐effect risk information to individual risk, preferring negatively framed side‐effect risk information.

Yeah and it's obvious that 97 and 99 look better than three or two. But in this case, if you want to know that it's not going to affect you, you would rather see the lower number than the bigger number. (FG2P6)

### Inclusion of benefit information

Benefit information included only in V2 went unnoticed by consumers. However, when prompted, consumers expressed that it gave them the rationale and confidence to take the medicine. Perceived benefit outweighing risk and potential improvement in quality of life increased willingness to commence treatment. Whilst autonomous consumers were willing to take ramipril in light of the benefit information, the magnitude was insufficient to shift some consumers' unwillingness to take ramipril. Additionally, consumers reliant upon their doctor for therapy decision making were unwilling to commence treatment unless deemed necessary by their doctor.

Although some wanted numerical benefit information, consumers acknowledged that benefit information without numerical figures led to increased perceived benefit of a medicine.

Because it could be 20% instead of 3%, they shouldn't put the percentage in it. (FG2P2)

## Discussion

A broad spectrum of consumer opinions was evident, where the majority preferred the simple tabular format in V1. The term ‘side‐effect’ was at times misconstrued. Additionally, confusion between ‘likelihood’ and ‘severity’ was prominent, where the majority had difficulty estimating the likelihood of experiencing side‐effects. Significant individual variation in risk appraisal was apparent, which may contribute to differences in consumer preferences for the alternative CMI versions. Specifically, a superior, preferred and correctly understood numerical descriptor did not emerge.

### Consumer perspectives – format and layout

The positive impact of the application of good information design, functional linguistics and medicine information expertise was evidenced by all consumers preferring at least one of the alternative CMIs, with positive consumer feedback provided on the revised layout. Due to the overwhelming preference for tabulated side‐effects (in V1 specifically), its feasibility in future WMI redevelopment and optimization should be considered. Earlier work has demonstrated that a redeveloped WMI leaflet for Mersyndol^®^ (Sanofi‐Aventis Australia Pty Ltd, Macquarie Park, Australia), which included a side‐effect table, performed better than the existing CMI when user tested to examine consumers' ability to locate and understand information.^34^ Furthermore, the findings of Tait *et al*.^41^ highlighted that tables presenting benefit and risk information promote the encoding of gist and verbatim representations in individuals with low literacy and numeracy, thus reinforcing the advantages of tabulating side‐effects and side‐effect risk information. However, Hawley *et al*.^42^ found that tables were only superior in promoting verbatim understanding. Accordingly, differences in table layout may account for differences in gist and verbatim understanding and should be explored in further detail.

### Side‐effects information

Consumers found it difficult to distinguish between a side‐effect, allergy and symptoms of a medical condition, where ‘likelihood’ and ‘severity’ were also used interchangeably. Health literacy, defined as ‘the ability to access, understand, evaluate and communicate information as a way to promote, maintain and improve health in a variety of settings across the life‐course’ (p. 11),^43^ is integral to an individual's ability to interpret and gain meaning from WMI,^44^ where sound health literacy is linked to increased awareness of the risk of experiencing medicine‐specific side‐effects.^45^ Consequently, literacy and/or health literacy issues that can affect consumer understanding must be considered in WMI development. For example, current CMI incorporates allergic reaction signs and symptoms within the side‐effects section, which may contribute to consumers' inability to distinguish between the two. A separate subheading for allergy information may be advantageous to promote vigilant monitoring, whilst preventing confusion, and may improve consumer health literacy.

### Understanding and opinions of numerical side‐effect risk information

Consumer misunderstanding of the non‐equivalence between percentages and natural frequencies is a major finding, which is integrated into risk appraisal processes. Accordingly, differences in consumer numeracy skills are also a possible key determinant in consumer risk appraisal, as highlighted in previous work.^46^ Furthermore, percentages and natural frequencies are not equivalent from a psychological standpoint, despite the equivalent numerical magnitude of risk.^33^ Thus, risk appraisal is not purely restricted to the type of information provided, but is also impacted by how information is presented. Interestingly, Brewer *et al*.^47^ have shown that females with higher health literacy had an increased understanding of various presentation formats used to convey numerical risk information. However, it must be noted that the present study did not quantitatively assess consumer numeracy in conjunction with health literacy. Accordingly, a quantitative examination of this potential interplay is needed to explore population health literacy and numeracy levels and their implications for appropriate consumer treatment side‐effect risk appraisal.

Many consumers could not gain meaning from numerical side‐effect risk information. A larger proportion of consumers preferred the absence of numerical information, which may be attributed to slight differences between the table formats and the overwhelming magnitude of numerical side‐effect risk information presented. FTT acknowledges that consumer knowledge of the exact numerical side‐effect risk may not be indicative of appropriate consumer risk appraisal/understanding and informed treatment decision making.^28^ Additionally, consumer preference for information presentation formats does not necessarily correlate with comprehension.^48^, ^49^ Interestingly, in a study conducted by Knapp *et al*.,^26^ consumers preferred side‐effect risk to be presented using percentages and frequencies in combination, where this approach did not yield worse estimates than using either alone. Evidently, the complexities inherent in the presentation of side‐effect risk information to consumers warrant further exploration.

Some consumer risk appraisal patterns were comparable with explanations provided by psychological theories. Consumer acknowledgement that natural frequencies are easier to understand is supported by FH. Differing consumer interpretation of risk highlights the role of numeracy in how consumers encode and appraise risk information via gist encoding, as explained by FTT.^29^ High numeracy^41^ and subjective numeracy^50^ have been shown to contribute to improved gist understanding. Interestingly, however, even though consumers acknowledged that the information represented a low numerical (verbatim or literal) side‐effect risk, some consumers still regarded it as high risk, which is in line with AH and the role that subjective perceptions have in forming consumer perspectives on risk. Consequently, a plethora of existing factors influenced risk perception.

Preferred numerical descriptors may diverge between consumers,^35^ and perceived risk may be dependent upon the numerical descriptor utilized. With natural frequencies, some consumers may picture patient cohorts and the inherent risk being communicated without difficulty,^51^ where this possible realistic dimension may contribute to fear and anxiety.^30^ However, the impact of affect‐laden images evoked from the use of natural frequencies upon accurate risk appraisal is context dependent^52^ and should be explored in further detail.

Additionally, information framing played a role in consumer understanding of side‐effect risk. Consumers who could interconvert between positively and negatively framed numerical side‐effect risk information with ease were more adept at ascertaining side‐effect incidence, regardless of how the information was framed. This finding is in agreement with the notion that high numeracy decreases susceptibility to framing effects.^53^

Importantly, consumers question numerical risk information reliability when small denominators are utilized.^51^ Regardless of denominator size, results of the present study indicate that consumers want to ascertain the test population size. It follows on that consumers' interest lies in ascertaining the likelihood of experiencing side‐effects and their own individual risk, which may be influenced by previous experiences of side‐effects, perception of health status and understanding of numerical side‐effect risk information, amongst other factors. Therefore, negative framing may assist consumers in ascertaining individual risk, as they are accustomed to receiving negatively framed side‐effect risk information. However, the non‐existence of ‘neutral framing’^54^ indicates that the intention and motivation behind WMI development is critical when addressing consumer information needs and promoting appropriate understanding. Consumer differences inherent in risk appraisal will also play a role in preferences for presentation of side‐effect risk information, where a ‘one‐size fits all’ approach may not be feasible.

### Inclusion of benefit information

Benefit information incorporated in V2 was overlooked by consumers, which may have been due to: minimal inclusion and emphasis on benefit information in comparison with the side‐effects information and its integration into the side‐effects section. Echoing gist reasoning in FTT, many preferred positive information without relevant numerical information, as it is sufficient to establish a benefit profile for the medicine (gist). It must be stressed that the inclusion of benefit information within the side‐effects section is not the sole, exhaustive method. The development of a separate benefit information WMI section may be more beneficial, promoting balanced decision making.

## Limitations

As this study aimed to qualitatively explore consumer opinions and understanding, the results are not generalizable. Furthermore, numerical data availability restricted the inclusion of other descriptors and types of numerical side‐effect risk information. A medicine bias may also be inherent, as only Tritace^®^ and Plavix^®^ brand CMIs were revised. However, arguably, the side‐effects sections were the focal point, rather than the study medicine itself. Also, this study was not completely accountable for the true behaviours of current medicine users, and the age‐specific inclusion criteria may have excluded the opinions of younger chronic medicine users. Additionally, consumer health literacy was not assessed, which limits the ability to draw conclusions on its impact on understanding of side‐effect risk information. Accordingly, this provides grounds for future work to be conducted in the area.

## Conclusions

WMI design and relevant side‐effects information play an influential role in consumer appraisal. Consumers prefer tabulated side‐effects in comparison with existing modes of presentation in CMI. Complexities in risk presentation emphasize that the format and visual assault of side‐effects information are important considerations. Numerical expression used to convey side‐effect risk information may fuel differing risk perception and understanding. Psychological models do provide insight into consumer risk appraisal processes. However, varied consumer opinions and interpretation of side‐effect risk information was apparent, where consensus was not reached on a preferred and well‐understood numerical risk descriptor. Future studies should endeavour to ascertain the concomitant impact of numeracy and health literacy upon balanced decision making and seek to address consumer misunderstandings. The challenge is to establish an optimal way to present side‐effect risk information to maximize understanding, by marrying consumer needs and understanding with what health‐care professionals, manufacturers and key stakeholders wish to convey.

## Financial support

None.

## Conflict of interest

David K Raynor is the co‐founder and academic advisor for Luto Research Ltd, a company that provides user testing services for health information.
